# Indicators of Family Care for Development for Use in Multicountry Surveys

**DOI:** 10.3329/jhpn.v30i4.13417

**Published:** 2012-12

**Authors:** Patricia Kariger, Edward A. Frongillo, Patrice Engle, Pia M. Rebello Britto, Sara M. Sywulka, Purnima Menon

**Affiliations:** ^1^Community Health and Human Development, University of California at Berkeley, Berkeley, CA, USA; ^2^University of South Carolina, Columbia, SC, USA; ^3^Cal Poly State University, San Luis Obispo, CA, USA; ^4^Yale University, New Haven, CT, USA; ^5^Food for the Hungry, Washington, DC, USA; ^6^International Food Policy Research Institute, Delhi, India

**Keywords:** Child, Development, Disciplinary measures, Family care index, Family care indicators, Parenting

## Abstract

Indicators of family care for development are essential for ascertaining whether families are providing their children with an environment that leads to positive developmental outcomes. This project aimed to develop indicators from a set of items, measuring family care practices and resources important for caregiving, for use in epidemiologic surveys in developing countries. A mixed method (quantitative and qualitative) design was used for item selection and evaluation. Qualitative and quantitative analyses were conducted to examine the validity of candidate items in several country samples. Qualitative methods included the use of global expert panels to identify and evaluate the performance of each candidate item as well as in-country focus groups to test the content validity of the items. The quantitative methods included analyses of item-response distributions, using bivariate techniques. The selected items measured two family care practices (support for learning/stimulating environment and limit-setting techniques) and caregiving resources (adequacy of the alternate caregiver when the mother worked). Six play-activity items, indicative of support for learning/stimulating environment, were included in the core module of UNICEF's Multiple Cluster Indictor Survey 3. The other items were included in optional modules. This project provided, for the first time, a globally-relevant set of items for assessing family care practices and resources in epidemiological surveys. These items have multiple uses, including national monitoring and cross-country comparisons of the status of family care for development used globally. The obtained information will reinforce attention to efforts to improve the support for development of children.

## INTRODUCTION

Family care practices during the first five years of life have a powerful influence on the rapid gains in children's motor, language, cognitive and socio-emotional development trajectories. These developmental domains lay the foundation for children's future development, behaviour, and functioning (1-4). Aspects of family care practices or qualities that have been commonly observed across cultures and appear to be fundamental to the caretaking of young children in a variety of cultural settings include responsiveness, warmth, provision and organization of the physical setting, and encouraging learning or exploration (1,2,5-7).

Measures of family care practices with global application are useful not only for understanding their influence on child development but also for guiding policy and intervention programmes aimed at improving the developmental trajectories of children the world over. There have been, however, no validated population-level indicators of family care practices for children's development. In response to this gap in measures, this paper describes the process of indicator development initiated in 2002 by UNICEF in three steps: (i) conceptualization of key constructs, (ii) assessment of quantitative and qualitative data from developed survey items in several countries, and (iii) recommendations for items to be included in surveys and further validation steps.

### Need for indicators

An indicator is based on a valid measure of a construct and has targets or levels defined, which suggest risk. Indicators are useful for a wide variety of purposes and are increasingly a requirement for international advocacy, action, and accountability. The process of developing an indicator requires several steps. The problem must be identified as important for key outcomes; there must be consensus of experts and practitioners on the definition of the construct; data are needed from a variety of cultural contexts to be sure that it is an adequate reflection of the construct across cultures; and it must be collected regularly and used by organizations and governments to direct policy and investment. To satisfy these criteria, an indicator is gradually refined on the basis of continuing data, and the validity of the indicator should be established using a criterion measure, such as observed behaviour.

Indicators are the currency that policy-makers use for having a better understanding of a topic that may be new or not well-documented, thereby increasing the likelihood of interventions to address that topic (8). This is a key issue for family care for development, which is not well-understood in many countries and may not be targeted for interventions because the absence of care is not recognized. Therefore, it is critical to develop useful indicators of family care for development.

The goal of this study was to develop a set of items that could be included in the Multiple Indicator Cluster Surveys (MICS) and nationally-representative household surveys developed by UNICEF to help countries evaluate progress toward achieving internationally-endorsed and supported goals relating to children's rights and well-being through examination of the risk and protective factors that influence child development. The surveys gather information on nutrition, health, education, water, sanitation, birth registration, and family care practices relating to health, nutrition, and hygiene and have been implemented by national governments of over 65 countries with strong technical support from country and regional offices of UNICEF, and the UNICEF MICS global team in New York City. The new items were to be directed toward children below 5 years of age because there are already child-specific survey modules for that age-group and were to be included in the third round of MICS administered in 2005-2006.

### Defining the construct of family care for development

Much of what is known about relationships between family care practices and child development comes from studies gathering extensive data on modest samples of children, often using observational techniques, both in developed (9-11) and developing countries (12-14). Such methods are not easily adapted to epidemiological studies or are useful for policy. Given the importance of family care in child development, it is imperative to develop measures and indicators of practices with universal appeal and applicability to assess whether families are providing their children with the psychosocial care that leads to positive development. The lack of global measures reflects the difficulty in identifying those specific aspects of family care that are most meaningful to measure cross-culturally and can be operationalized and measured at the population level (5).

Bradley and Caldwell (15) describe family care as “a set of environmental actions performed by a caregiver, or environmental conditions arranged by a caregiver that…allow a child to adapt and to pursue goals.” While providing conditions and opportunities in the environment, family care also helps regulate a child's psychobiological state so that a child can best take advantage of opportunities and experiences that promote positive development. Emanating from this conceptualization, a large number of studies in developing countries have used the Home Observation for Measurement of the Environment Inventories (HOME) (16).

The HOME assesses household support and stimulation provided to children during hour-long, naturalistic observation and interview sessions at the child's home. The four age-specified HOME inventories include scales measuring aspects such as responsiveness, acceptance (including discipline), provision of appropriate stimulation, and materials for encouraging learning/development, and the physical environment of the household (16), which align with dimensions of caregiving identified in the literature. Higher scores on the HOME–indicating greater support and stimulation–have predicted better child outcomes across a range of ages, ethnicities, and economic groups (14,17-25).

Reviews of cross-cultural research (17,26) suggest that the inventories represent some universal aspects of the home environment that are important for positive child outcomes. Items with the best validity and cultural equivalence were those measuring cognitive stimulation or learning rather than those assessing emotional support, possibly because the cognitive stimulation items are more specific and tangible than those assessing non-specific, abstract manifestations of family care where interpretations may be more easily affected by culture (27). Thus, the HOME scale became one of two conceptual bases for defining family care for development. However, while the HOME has been successful in assessing a number of aspects of the family care environment worldwide, the observation/interview method is time and labour-intensive and prohibits its use for providing measurement at a national level. Therefore, there has been a need for a set of items that could be used in an epidemiologic survey to represent family care for development at a population level and achieve the other goals of advocacy, action, and accountability.

A second conceptual basis for family care for development is specification in the UNICEF's conceptual framework of care for nutrition, which has been expanded to measure care practices that also influence child development (28). Care is “the provision in the household and the community of time, attention and support to meet the physical, mental, and social needs of the growing child and other household members” and includes six categories, including psychosocial care.

The capacity for such family care for development is, in turn, dependent on the availability of resources for caregiving at the household level (28). Three sets of resources available to the caregiver (29) have been identified as (i) human resources, including caregivers’ knowledge and health (30), and fathers’ participation in caregiving (31-32); (ii) economic resources (33-34); and (iii) organizational support, such as the availability of appropriate alternate caregivers as needed (35).

Resources empirically related to family care behaviours and/or child outcomes in some studies include parenting knowledge or beliefs (36-38), caregiver depression (39-41), socioeconomic status (SES) (18,34), and father's involvement (42). Although much of this research has taken place in developed countries, some work has been done in developing countries. For example, maternal depression was associated with poor nutritional status of children in India and Viet Nam but not in Peru and Ethiopia (43). Hence, there is a need for measuring these resources globally and examining how these relate to family care behaviours and child outcomes worldwide.

## MATERIALS AND METHODS

### Overview of indicator development

A mixed method (quantitative and qualitative) design (44) was employed to gather different types of information about the items tested. Qualitative methods included the use of expert panels to identify and evaluate the performance of each candidate item, and informant interviews and focus-group discussions were used in the field to learn how well the items were understood. The quantitative methods ensured adequate variability of items within and across countries and evaluated associations with presumed correlates, such as SES, mother's literacy, and nutritional status in the three countries in which these were available. Limitations of time and funds precluded validation with measures of cognitive development but this has been done in Bangladesh (45). A final set of items was selected and incorporated into the MICS.

### Phase I: Theoretical conceptualization and identification of domains and items

In November 2002, UNICEF convened a panel of 25 international experts (Expert Panel I) with expertise in human development, anthropology, nutrition, and measurement to (a) develop a framework of domains of family care practices and resources important for young children's development, (b) evaluate possible items to use in pilot testing, and (c) define priorities for testing. The selected family care domains and items were culled from the multidisciplinary literature on the practices and resources identified as important for the motor, social, emotional, cognitive, and language development of young children, and caregiver resources.

The majority of candidate items were selected from instruments that have shown good psychometric properties across a variety of samples in the USA (e.g. HOME; Early Childhood Longitudinal Study measures, see www.nces.ed.gov/ecls/; National Household Educational Surveys, see www.nces.ed.gov/nhes/) or in developing counties (46-48). Where no suitable candidate items could be found in the literature, these were suggested by panel members with expertise in that domain.

### Phase II: Field-testing and informant interviews

During the spring and summer of 2003, the items were field-tested in Brazil, Burkina Faso, Nepal, Uganda, and Zanzibar (United Republic of Tanzania), representing a variety of cultural contexts but using the existing projects or programme infrastructure. Data on SES, maternal education, and nutritional status were also available from these existing projects for Nepal and Zanzibar. Informant interviews on the items were conducted in Bangladesh, Jamaica, and Mexico as well.

Informant interviews, a qualitative method for evaluating how questions and responses sets are interpreted by people representative of the population of interest—and whether the interpretations reflect the intended purpose of the items (i.e. content validity) (49-51)—were conducted in convenience samples in Bangladesh (n=10 mothers), Jamaica (n=10 mothers), and Mexico (n=30 mothers). Using concurrent verbal probing, informants were asked to answer a question, and then asked more specifically about the item and response set to gather more information about the bases for their replies. The probes were designed to assess informants’ comprehension and interpretation of the item, their confidence in their responses, and how they remembered the information used for responding to the question. Interviews were either recorded and later transcribed, or noted and later summarized.

The quantitative data were collected with an orally-administered survey. In each locale, the survey items were translated by local staff. After fieldworkers were trained on the measures, the questionnaire was pre-tested and administered in Brazil (n=50; age 1-81 months), Burkina Faso (n=119; 0-56 months), Nepal (n=564; age 17-31 months), Uganda (n=2157; ages 0-36 and 37-60 months), and Zanzibar (n=807; age 18-35 months). In Brazil, participants were from Canudos, Bahia, in an urban area where about half of adults receive primary and about one-third secondary education. In Burkina Faso, mothers were recruited from Zondoma province in the north, a rural, poor, semiarid, subsistence-farming area with mixed monogamous and polygamous families. In Nepal, mothers were from a rural, poor, subsistence-farming area in the southeast. In Uganda, the questions were asked as part of an evaluation of a programme funded by the World Bank on nutrition and early child development, assessed in 2003; the sample consisted of households from five districts in the eastern and central areas of Uganda, which are primarily Luganda. In Zanzibar, participants were from poor, rural and peri-urban areas on Pemba Island where the main occupations are fishing and farming, and malaria and other parasites are endemic.

Descriptive statistics for responses to each item were calculated by site, and for the three more complete datasets, chi-square analyses were conducted comparing level of SES with item responses. These data were used for examining whether response patterns showed discrimination within and among the sites as would be expected for some items, as a way of indicating both convergent and discriminant validity (44). SES was represented by a composite score, based on the measurement of a variety of personal (e.g. parental education, literacy, income) and environmental (e.g. house quality, access to water) factors that were previously successfully used in other analyses utilizing the same datasets (52-53). Higher and lower SES were indicated by splitting the samples at the median SES score. Two SES groups were used for ease of comparison and display; the use of more graduated SES groups (i.e. terciles or quartiles) did not change the patterns of results.

### Phase III: Item selection and indicator creation

In November 2003, a panel of 27 experts was asked to finalize the selection of items to assess the quality of family care for development in the context of the MICS questionnaire, based on the qualitative and quantitative information. Data on each item were examined both within and between the country samples to ensure that each item showed ‘measurement equivalence’, or evidence that the item had the same meaning and was understood the same way in different cultures (54). Items were evaluated according to the clarity of the question, the quantitative data and, finally, for the value of the item for influencing policy or monitoring programming (Table 1).

## RESULTS

Results are divided in three sections: (i) identification of candidate items for field-testing (Phase I); (ii) findings from the field-testing (Phase II), and (iii) evaluation of the suitability of candidate items and finalized items and indicators recommended for inclusion in MICS surveys (Phase III).

### Phase I: Item identification

Expert Panel I defined seven family care domains and seven caregiving-resource domains as derived from the HOME scale and the UNICEF's conceptual framework as having global applicability. The domains of family care were quality of verbal interactions, support for learning, limit-setting (i.e. disciplinary) techniques, consistency of support, support for emotional well-being and acceptance, support for sense of self, and responsiveness to the child. The domains of resources included four categories at the level of the caregiver (caregiver's stress, caregiver's time availability, physical health, and knowledge) and three at the household level (family cohesion/functioning, social support, and organization of the care environment).

**Table 1.** Criteria used for evaluating the performance of the candidate items

1. Conceptual basis	2. Performance	3. Discrimination	4. Usefulness
1a. Is the concept being measured important in a certain group?	2a. Was the item clear and interpreted by the respondent as it was intended?	3a. Do the responses vary within groups?	4a. Do the results convey information that will be meaningful and useful to programme and policy staff and officials for purposes, such as advocacy, planning, decision-making, and early intervention?
1b. Does the item measure the concept adequately?	2b. Did the pre-specified responses to the questions make sense to the respondent?	3b. Do the responses vary among groups in ways that might typically be expected?	4b. Can the results be combined with that of other questions to make the information more meaningful and useful?
1c. Does the item measure the concept similarly across groups?	2c. Did the back-translation from all the countries indicate that the same wording was used in all places for the question and the response (where the same versions were tested)?		
1d. Does the question and the response set provide a basis to discriminate between children who are better or worse-off for that particular domain of caregiving?	2d. Did the group in which the item was administered influence the responses and interpretation of the question (i.e., did it perform similarly across groups)?		

With advice from Expert Panel I, these 14 domains were combined into four family care domains: responsiveness and acceptance, support for learning/stimulating environment, limit-setting techniques, and caregiver responsiveness during feeding and three resources domains: availability and use of alternate caregivers, father's involvement with child, and maternal depression symptoms. Responsiveness during feeding was of interest because it could be another measure of responsivity and overlapped with nutrient intake but because it may not relate theoretically to a child's development, it is not considered further in this paper. The final 27 items are shown in Table 2 and were presented to the second panel.

### Phase II: Informant interviews

The content analyses of the informant interview data examined two issues: whether the items were clearly expressed and, second, whether the item was relevant and appropriate in each country. It was unclear what defined an ‘adult’ for item number 203, 205, and 401 (Table 2), and items asking about books and play-materials needed more clarification about which types of books and play-materials could be included as valid responses. Feedback from all sites indicated the limit-setting item, “What do you usually do when your child does something you don't like?” needed more parameters around the behaviours being questioned (e.g. fussing, being naughty, engaging in unsafe activities) and also needed to be altered so that multiple limit-setting techniques could be selected. Items needing simplification were those asking respondents to recall information for the last week (item 205) or month (items 701-707); it was recommended that the timeframes for the items be shortened to make it easier for respondents to answer accurately.

**Table 2.** Candidate items

**Responsiveness and acceptance (Yes/No)** 101. Children seem to demand attention when their parents are busy, doing housework, for example. Do you usually respond to your child's demands for attention while you are working? 102. Has your child done anything in the last week that pleased you very much? **Support for learning/stimulating environment (*Circle the best answer*)** 201. About how many children's books or picture books do you have for (CHILD)? None 1-2 3-5 6-9 10-19 20 or more 202. How many other books are there in the household? None 1-2 3-5 6-9 10-19 20 or more 203. Children play with a lot of different things. What kinds of things do you have that children play with? (*Read list; indicate ‘yes’ or ‘no’ for each*) Toys made by an adult Household objects Materials from outside the house Toys that make music Toys for building things Things for drawing and writing Things for moving a lot, like balls, rattles, bat, hopping rope Toys for pretending like dolls, sticks for animals, pretend cups, plates Other things child plays with ___________. 204. About how many hours a day does your child usually watch TV? [_____] 205. In the past week, on how many days did you or any other adult family member do the following with (CHILD)? Read books or look at picture books Tell stories Sing songs Go to the market or store, or visiting outside the home Play Spend time in learning activities, like counting, naming objects, and drawing Do household chores with, like cooking, cleaning, caring for animals Teach about spiritual or religious practices Sit with the child during the main meal of the day Feed or assist the child to eat Talk during meals **Setting limits** 301. When your child does something that you do not want him or her to do, what do you usually do? (*Circle the best answer–do not read*) Nothing; ignore him/her Limit his/her movement Slap hand when child touches something Tell ‘no’ and expect to obey Tell ‘no’ and explain why Have child sit down or go to other room for quiet time Shout at him/her Put things out of reach Distract with activity Take child away Other________ 302. Sometimes, children behave pretty well and sometimes they don't. On how many days, if any, have you had to hit your child in the past week? [_____] **Alternate care situation** 401. When you have the leave the house to go for shopping, washing clothes, or for other reasons, what do you usually do with (CHILD)? (*Circle the best answer*) Take with me Leave with grandmother Leave with other adult relative Leave with other adult Leave with sibling/child ≥10 years old Leave with sibling/child < 10 years old Alone, if I can't find anyone else to watch child 402. On how many days per week do you usually leave (CHILD) with someone else? [_____] 403. On those days, about how many hours are you usually away? [_____] **Father's involvement (*Indicate ‘Yes’ or ‘No’ for each*)** Does (CHILD'S) father usually: 501. Contribute money to support the child, paying for food or school? 502. Play and talk with the child? 503. Feed and care for the child? 504. Hold and carry the child? 505. Teach things to the child? **Responsive feeding (*Circle the best answer*)** 601. How do you know when your child is hungry? Cries Asks for food, points, or uses gestures (but does not cry) Other __________________ 602. When you serve (CHILD) food, how is it served? Separate bowl Common or shared family plate Child has not started eating other foods 603. What do you usually do to get your child to eat? Nothing Tell child to eat Encourage, praise, play, or hold Give other types of food Force, threaten, or hit Other _________________ **Depression symptoms** For each of the following questions, think about how you have felt over the past 4 weeks. (*Indicate Yes/No*) 701. Do you feel tired all the time? 702. Do you find it difficult to make decisions? 703. Is your daily work suffering? 704. Do you have trouble thinking clearly? 705. Do you find it difficult to enjoy your daily activities? 706. Have you had days when you felt sad or unhappy for most of the day? 707. Have you had days when you lost interest in most things, like work or other things you enjoy doing?

Regarding relevance and appropriateness in countries, the informant interviews suggested that cultural or lifestyle factors affected the performance of items relating to acceptance of the child, father's involvement, and maternal depression. Item 102, “Has your child done anything in the last week that pleased you very much?” was easily understood and answered in Jamaica and Mexico. In Bangladesh, however, the concept was not understood as it was intended, with some caregivers explaining that their child pleased them because he/she was healthy. In Jamaica, it was believed that the social desirability of responding positively to items regarding the father's role in caregiving (items 501-505) may obscure honest responses while in Mexico it was questioned how to administer these items when biological fathers were absent or other father figures were present. In Bangladesh, the father-related questions were fairly and easily understood.

Performance of the maternal depression symptom items varied across the three sites. In Mexico, women understood the items but expressed difficulty responding to them as they were not accustomed to speaking about their feelings. Items 702-705 were not readily understood in Bangladesh. In Mexico and Jamaica, it was intimated that item 705 “Do you find it difficult to enjoy your daily activities?” was not a valid symptom of depression as it was generally accepted that most women's work was not something to be enjoyed. Across all three sites (i.e. Bangladesh, Jamaica, and Mexico), the most well-understood items were those pertaining to the activities adults did with children and whether the child was hit during the past week.

### Phase II: Field-testing

Frequency analyses for the items for Brazil, Burkina Faso, Nepal, and Zanzibar are shown in Table 3. The response variability was used by Expert Panel II, in conjunction with the criteria in Table 1, to assess the performance of the items.

The majority of items showed variability in responses, both within and among sites, suggesting these were potentially useful for differentiating practices of families in different conditions (Table 3). Items with little variability included those on acceptance and responsiveness; father's involvement; item 602, “When you serve your child food, how is it served?”; and item 701 “Do you feel tired all the time?” The other depression symptom items (items 702-707) generally showed some discrimination within countries but less discrimination among countries.

Within-sample variability of items by SES was examined in both Zanzibar and Nepal, using chi-square analyses (Table 4). For items assessing support for learning/stimulating environment, availability and use of alternate caregivers, strategies to encourage eating and depressive symptoms, there was a tendency for higher SES to be associated with more positive family care practices or resources in both sites. This corroborates previous work examining relationships between SES and the family care environment (34,55-56) and SES and depression (57-58). SES was not associated with caregivers’ propensity to respond to their child's demands for attention (item 101) or whether they hit their child in the past week.

Some differences among sites were also found. Nepalese caregivers with higher (vs lower) SES were more likely pleased by something their child did in the past week and to use more positive limit-setting strategies. In Zanzibar, higher (vs. lower) SES caregivers more often reported that their children used words or gestures to indicate their hunger and that fathers were usually involved in a range of care practices with their child. These differences were not consistent across cultures.

### Phase III: Item selection and indicator creation

To evaluate suitability of items for inclusion in the MICS surveys, Expert Panel II reviewed findings from both informant interviews and the quantitative data, using the criteria: (a) theoretical clarity, (b) clarity of the questions and concepts, (c) reasonable pattern of variability across and within countries, (d) consistent associations with criteria across countries, (e) usefulness for policy advocacy and accountability, and (f) appropriate across the age range of 0-59 month(s).

Finally-selected items from four domains were: play-activities with adults, the availability of books and play-materials that promote development, limit-setting, and the availability and use of alternate caregivers. Items not selected were: acceptance and responsiveness and the father's role in caregiving. The panel agreed, however, that some information about father's involvement in caregiving could be measured by specifying the adult participating in learning activities with the child.

There was concern about the assessment of maternal depression symptoms. The panel finally recommended a set of items from the Centers for Epidemiological Studies Depression Scale (CES-D) (59), which focuses on emotionality and mood as these items have discriminated between high and low levels of depression symptoms (60). The CES-D has been studied in many countries (for example, 61-63).

Subsequent to the meeting, the UNICEF staff adapted a measure of limit-setting (i.e. child discipline) that was based on the WHO World Safe survey from 16 countries (64-67), which was a slight adaptation of the items recommended by the panel. The depression items were not included in the final MICS survey due to ethical concerns that identifying depression would require a referral for interventions, and there was concern that asking about women's feelings would be a different role for the interviewers to which they might not be able to switch. All of the other recommended items were adapted and used in the 2005-2006 MICS. Six play-activity items were in the Core Module, and the books and play-materials, alternate care, and limit-setting items were in the Optional Module. Fifty countries used the activity items, and the majority used at least some of the optional items (see information about MICS3 at www.childinfo.org).

**Table 3.** Response frequencies (%) to administered items by site

Item	Response	Brazil (N=50)	Burkina Faso (N=99)	Nepal (N=527)	Zanzibar (N=807)
Responds to demands	Yes	82.0	98.0	96.2	98.0
Pleased by child	Yes	64.0	47.5	70.3	83.6
Children's books	None	46.0	-	85.9	88.8	
	1-5	32.0	-	11.4	10.9	
	≥6	22.0	-	2.60	00.2	
Other books	None	28.0	-	44.0	88.8	
	1-5	26.0	-	23.8	10.9	
	≥6	46.0	-	32.2	00.2	
Play-materials	Made by adult	46.0	-	-	58.4	
	Household objects	68.0	-	-	99.0	
	Outside materials	34.0	-	-	94.9	
	Musical toys	28.0	73.5	24.4	40.6	
	Building toys	20.0	69.4	16.8	44.1	
	Drawing/writing	48.0	52.0	15.2	59.3	
	Toys for moving	56.0	82.7	64.0	89.2	
	Toys for pretending	54.0	72.4	39.4	91.8	
	Other	22.0	-	65.7	2.4	
Daily TV watching (hr)	0	38.0	-	63.0	71.7	
	1-2	40.0	-	32.8	23.7	
	≥3	22.0	-	4.0	4.6	
Activities with adults	Read/look at books	26.0	-	25.7	42.2	
(≥1 day)	Tell stories	46.0	12.2	14.1	38.2	
	Sing songs	56.0	17.3	36.2	76.4	
	Go outside home	64.0		51.2	94.8	
	Play	86.0	66.3	67.6	97.9	
	Learning activities	52.0	-	42.3	78.8	
	Household chores	30.0	-	63.2	93.6	
	Religious practices	60.0	-	34.9	76.8	
	Sit with child during				93.5	
	meal	-	87.8	-	24.2	
	Feed/assist child to eat	-	-	-	90.8	
Setting limits^a^	More positive strategies	36.0	-	24.1	7.4	
	Less positive strategies	54.0	-	67.6	54.1	
	Other	10.0	-	8.4	38.0	
Days child hit, past week	0	56.0	-	29.9	33.7	
	1-2	36.0	-	39.4	32.7	
	3-7	8.0	-	42.1	33.6	
Alternate caregiver^b^	None, take child with me	36.0	40.4	13.7	7.7	
	Adult	50.0	32.3	42.1	28.9	
	Child ≥10 years old	10.0	23.2	17.0	45.8	
	Child <10 years old	2.0	3.0	20.8	16.1	
	Alone, if none available	2.0	0.0	2.1	1.1	
Days per week with	0	34.0	-	0.0	12.3	
alternate caregiver	1-4	44.0	-	53.6	45.7	
	5-7	22.0	-	45.9	41.9	
Hours per week with	0-0.5	32.0	-	1.6	9.5	
alternate caregiver	1-4	54.0	-	72.5	54.0	
	≥5	14.0	-	25.2	33.3	
Father usually…						
…contributes money	Yes	78.0	83.7	96.6	95.0	
…plays/talks with child	Yes	70.0	84.7	91.4	91.9	
…feeds/cares for child	Yes	68.0	84.7	90.5	90.1	
…holds/carries child	Yes	76.0	79.6	92.0	91.8	
…teaches child	Yes	62.0	55.1	71.4	90.0	
Child indicates hunger	Cries	14.0	-	16.8	35.8	
	Asks, gestures	80.0	-	83.0	57.4	
	Other	6.0	-	0.2	6.8	
Child feeding	Separate bowl	62.0	-	98.5^c^	71.2	
	Shared bowl	28.0	-	91.4	28.6	
	Not eating food yet	10.0	-		0.2	
Encourage eating^d^	Nothing/tell child to eat	28.0	-	66.8	27.5	
	Encourage, praise	68.0	-	29.1	20.4	
	Force, etc. Nothing/good appetite	2.0 -	—	1.9 -	5.9 42.0	
	Other	2.0	-	2.1	4.1	
Tired all the time	Yes	70.0	80.9	67.6	77.4	
Difficulty making decisions	Yes	50.0	-	48.6	54.3	
Work suffering	Yes	50.0	-	41.1	60.3	
Trouble thinking clearly	Yes	48.0	-	46.3	40.0	
Difficulty enjoying daily activities	Yes	32.0	-	40.0	60.9	
Sad/unhappy	Yes	66.0	45.2	55.4	56.8	
Lost interest	Yes	50.0	15.7	47.6	53.3	

^a^More positive strategies=responses 2,5,6,8-10; Less positive strategies =1,3,4,7

^b^Adult=responses 2-4

^c^More than one response possible

^d^Responses 1-2 combined, responses 3-4 combined

## DISCUSSION

This project resulted in a set of globally-applicable items intended for use in household surveys to assess family care for development of young children. The project rationale was that valid population-based indicators of family care practices and resources that promote the motor, socio-emotional and cognitive development of children would provide much-needed evidence on the proximal contexts for early child development, thereby spurring attention and investment in supporting families in caregiving. The recommended items were included in the MICS3 (2005-2006) for 50 countries and in the MICS4 (2009-2013) for 57 countries.

The recommended items assess two practices: support for learning/stimulating environment and limit-setting techniques, and one caregiving resource: availability and use of alternate caregivers. The neurobiological literature recognizes psychosocial and cognitive stimulation as strong positive influences on early brain development, and abuse, violence, and neglect as negative influences. Positive psychosocial stimulation is often linked with the availability of resources (68-70). The selected items represent three of the six domains of the HOME scale. These domains and their importance to development of young children are understood to be universal (27). This study provides evidence that the selected items assess these domains in a comparable way across countries. The domains not represented are warmth, responsiveness, and quality of the physical environment, although some of these are tapped by the items on the alternate caregiver. Quality of the physical environment was difficult to assess cross-culturally through the questionnaire items.

The Convention of the Rights of the Child (71), the most widely-ratified human rights treaty and one that influences country-level policies and programmes, highlights the role of the family in upholding children's rights to survival, development, and protection (72). The recommended items are consistent with both international standards and the academic literature, aligning with child survival, development, and protection and also with positive and negative influences on early development. These items are applicable to household-level surveys that provide data for national policy and programmes and will provide global indicators for comparisons across countries. The data resulting from these items will provide useful information to countries on aspects of caregiving that need support and improvement while upholding national commitments to child rights. Such data have not been available; consequently, family care practices have not been measured and not given their due attention by policies and programmes.

**Table 4.** Response frequencies (%) for items by SES category, Zanzibar and Nepal

Item	Response	Zanzibar		Nepal	
		Lower SES	Higher SES	Lower SES	Higher SES
		(N=381)	(N=425)	(N=242)	(N=270)
Children's books	None	93.4^a^	84.7	93.9^a^	78.7
	1-5	6.0	15.3	5.7	16.5
	≥6	0.5	0.0	0.4	4.8
Other books	None	6.6^a^	3.5	61.6^a^	43.9
	1-5	66.7	58.7	24.1	24.0
	≥6	26.8	37.8	14.3	32.1
Play-materials	Made by adult	26.7	31.7	-	-
	Household objects	47.0	52.0	-	-
	Outside materials	44.0	50.9	-	-
	Musical toys	15.0^a^	25.7	10.1	14.5
	Building toys	19.1^a^	25.0	6.6	10.3
	Drawing/writing	25.2^a^	34.1	3.9^a^	11.4
	Toys for moving	41.7	47.5	26.7^a^	37.5
	Toys for pretending	43.0	48.8	18.6	20.7
	Other	-	-	-	-
Activities with adults (=1 day)	Read/look at books	17.5^a^	24.7	6.8^a^	19.0
	Tell stories	16.7	21.5	3.9^a^	10.1
	Sing songs	35.9	40.6	15.1	20.9
	Go outside home	44.5	50.3	23.2	27.9
	Play	46.2	51.7	29.8^a^	37.5
	Learning activities	36.7	42.1	17.0^a^	25.1
	Household chores	43.9	49.6	26.3^a^	36.4
	Religious practices	35.2	41.6	11.8^a^	22.6
	Sit with child during meal	-	-	-	-
	Feed/assist child to eat	-	-	-	-
	Talk during meal	-	-	-	-
Setting limits^b^	More positive strategies	5.0	5.9	11.9^a^	20.0
	Less positive strategies	95.0	94.1	88.1	80.0
Days child hit, past week	0	32.8	34.6	29.0	30.4
	1-2	30.4	34.8	26.5	27.8
	3-7	26.7	23.5	43.3	41.9
	≥8	10.0	7.1	1.2	0.0
Alternate caregiver^c^	Adequate	36.9^a^	45.8	33.7^a^	42.4
	Inadequate	10.5	6.8	14.3	9.5
Hours per week with alter-	0-10	20.1^a^	29.7	13.6	19.7
nate caregiver (402 and 403)	11-25	15.1	11.0	14.8	15.9
	>25	12.0	12.2	18.9	17.1
Father usually…					
…contributes money	Yes	92.7^a^	97.2	94.3	98.2
…plays/talks with child	Yes	89.0^a^	94.6	90.6	91.9
…feeds/cares for child	Yes	87.1^a^	92.7	89.8	90.9
…holds/carries child	Yes	89.0^a^	94.4	91.4	92.6
…teaches child	Yes	86.4^a^	93.2	64.1^a^	78.3
Child indicates hunger	Cries	42.3^a^	30.0	18.4	15.4
	Asks, gestures	52.2	62.0	81.6	84.2
	Other	5.5	8.0	0.0	0.4
Child feeding	Separate bowl	75.2^a^	67.7	-	-
	Shared bowl	24.5	32.3	-	-
Encourage eating^d^	Nothing/tell child to eat	31.0^a^	26.8	69.4	64.0
	Encourage, praise	15.7	24.6	26.5	32.0
	Force, etc.	3.9	7.7	2.9	1.1
	Nothing/good appetite	47.2	37.3	-	-
	Other	2.1	3.5	1.2	2.9
Tired all the time	Yes	79.3	75.8	72.2	63.6
Difficulty making decisions	Yes	59.8^a^	49.3	53.9^a^	43.8
Work suffering	Yes	65.1^a^	56.1	48.2^a^	34.6
Trouble thinking clearly	Yes	46.7^a^	34.0	50.6^a^	41.9
Difficulty enjoying daily activities	Yes	64.6^a^	57.7	50.2^a^	30.9
Sad/unhappy	Yes	62.7^a^	51.4	64.9^a^	47.1
Lost interest	Yes	57.2^a^	49.9	51.0	45.2

^a^Chi-square analyses significant at p<0.05;

^b^More positive strategies=responses 2, 5, 6, 8-10; Less positive strategies=1, 3, 4, 7;

^c^Adult=responses 2-4;

^d^Responses 1-2 combined, responses 3-4 combined

The use of a mixed-method approach both broadened and deepened the project's capacity to produce a recommended set of items. This project employed global, regional and national expertise while combining qualitative and quantitative data. The process of indicator development reported here is the first step. Further work is needed, using the data from the participating countries (data available at www.childinfo.org). The items need to be validated against a criterion of child development. To date, this has been done in Bangladesh (45) where responses to the family care questions were related to scores on the Bayley Scales of Infant Development II (73) and a language comprehension and expression scale, based on the MacArthur Inventory (74), among 757 children aged 18 months. The results showed strong correlations between the variety of play-materials, play-activities, reading materials items, and the language scores. Also found were lower but significant correlations with the motor, mental and behaviour scales of the Bayley. The maternal depression scale was significantly and negatively associated with mental development, language expression, and some indices of the behaviour rating scale, although these correlations were low. These associations persisted even after controlling for SES. This study suggests that the items have good validity for predicting concurrent mental, motor and behaviour development as assessed by standardized tests in toddlers.

Eventually, a version of these items should be able to be used in epidemiological studies and programme evaluations to assess characteristics of family care relating to developmental outcomes. Such information is crucial, not only for understanding how household environments might be improved but also for advocating with national governments to increase investments in family care for child development. Ultimately, such data could be used for informing international policy-makers concerned with improving the lives of families and children. The availability of these items will provide much-needed information about the status of family-care settings globally and that this information will reinforce attention to efforts to improve support for families in supporting their children's development.

## ACKNOWLEDGEMENTS

Authors are thankful to the many people who contributed to this project and the people who participated in the expert panels.
